# Editorial: Deprescribing and Minimizing Use of Anticholinergic Medications

**DOI:** 10.3389/fphar.2021.820051

**Published:** 2021-12-15

**Authors:** Roy L. Soiza, Malaz A. Boustani, Noll L. Campbell, Arduino A. Mangoni

**Affiliations:** ^1^ NHS Grampian and Ageing Clinical and Experimental Group, University of Aberdeen, Aberdeen, United Kingdom; ^2^ Regenstrief Institute, University of Indiana, Bloomington, IN, United States; ^3^ College of Medicine and Public Health, Flinders University, Adelaide, SA, Australia

**Keywords:** anticholinergic, antimuscarinic, prescribing, drug safety, medication withdrawal, medicines optimisation

The global ageing population is increasingly challenged by multi-morbidity resulting in polypharmacy (Soiza 2020). Over the last 2 decades, medications with anticholinergic properties have been particularly strongly associated with a wide range of adverse outcomes (Lowry et al., 2011; Stewart et al., 2021). Despite this, anticholinergic medications have a broad range of indications and continue to be widely prescribed (Bostock et al., 2010). The present challenge is to consider how best to minimize harm from this group of drugs. There is a growing evidence base for the safety and efficacy of deprescribing approaches. However, deprescribing approaches to anticholinergic therapy specifically remains relatively understudied. Although there are now multiple tools to measure the extent of anticholinergic prescribing, it is unclear what tool performs best (Hanlon et al., 2020). Moreover, there is currently no simple or reliable way for patients to know if their medications have anticholinergic effects. There are significant evidence gaps around how best to optimize use of these medications, how to usefully involve key stakeholders in decisions around their medications or what deprescribing interventions should look like. The effects of deprescribing interventions on key outcomes of patient safety and experience are unknown. Nevertheless, public and clinician awareness of the dangers of anticholinergic therapy is growing and this is reflected in the increasing amounts of research on anticholinergic medications and deprescribing opportunities. It is likely the next decade will see major new research filling much of the evidence gap and significant improvements in the safety of prescribing medications with anticholinergic effects. This special edition of *Frontiers in Pharmacology* aimed to present the state of the art in responsibly prescribing and deprescribing anticholinergic therapy.

A comprehensive systematic review and meta-analysis by (Graves-Morris et al.) found anticholinergic burden is associated with an increased risk of death, odds ratio of around 1.4, in those aged 65 or over. This is important because previous systematic reviews reported contrasting results on the association between anticholinergic burden and mortality (Fox et al., 2014; Ruxton et al., 2015). As well as including more recent studies, the present review focussed on studies recruiting older people, in whom higher anticholinergic burden is more likely to lead to death due to lower resilience. Interestingly, the association was present and of a similar magnitude even where anticholinergic burden was “low.” The review included studies enrolling almost half a million people and also sought to clarify if any particular measure of anticholinergic burden might be superior for the purposes of risk prediction. It found a larger evidence base for the Anticholinergic Cognitive Burden scale (ACB) (Boustani et al., 2008) but could not make recommendations on superiority of any measure due to a lack of studies comparing various measures. This work further adds to the evidence for anticholinergics to cause serious harm, though it should be noted the association may not be causal and the authors found most studies had a high risk of bias, with high heterogeneity in study design and reporting. Ultimately, only randomised controlled trials of deprescribing or anticholinergic avoidance strategies may prove that avoiding or minimising anticholinergic use saves lives.

The challenges of designing such trials are neatly illustrated in the study by (Cunningham et al.). Patients want to be engaged in decisions around their medications, and interventions will need to factor this in before they can even be trialled. Patients want to be clear about the reasons for reducing their anticholinergic burden to avoid misconceptions around the motivation for reducing it. The importance of good communication was manifested by their desire to have a point of contact throughout the life of the trial, suggesting there are underlying concerns about the safety of deprescribing approaches but also a clear willingness to try out such approaches if any concerns can be answered. Healthcare professionals were concerned about workload and lines of responsibility. Pharmacists were felt to be best placed to undertake reviews of anticholinergic burden, but overall responsibility was felt best placed with GPs. This shows the importance of a team approach and excellent communication between GP, pharmacist and patient. Any intervention will need to embed this team approach or be likely to be doomed to failure. The better news was that willingness to participate in trials of deprescribing interventions was high, perhaps reflecting increasing awareness of the dangers of anticholinergic prescribing and an acceptance that current practice may not be in patients’ best interests.

The lack of clear consensus on how best to measure anticholinergic burden is the focus of work by (Dihn et al.). Their protocol for a study in the older general practice population will investigate whether existing measures are able to predict falls in this group. In the expectation that existing measures could be improved upon, they will derive and validate a new measure of anticholinergic burden specifically for the prediction of falls. It is entirely credible that different anticholinergic burden scales may perform better for predicting different outcome measures and their precise calibration for prediction of clinically important events is uncertain.

Most of the focus of anticholinergic burden quantification and reduction has come from studies in North America, Europe and Australasia. However, a reminder that this is a global phenomenon comes from a new study by (Fadare et al.). They found a third of older ambulatory patients in a tertiary referral centre in Nigeria had routine exposure to anticholinergic medications. As many as 15% had high levels of exposure. Unsurprisingly, anticholinergic prescribing was associated with polypharmacy and multi-morbidity suggesting these groups could be the focus of any anticholinergic deprescribing interventions.

Finally (Elliot et al.) remind us of the human story behind concerns over anticholinergic prescribing. They present a case report that neatly illustrates the dangers to patients, especially where there is multi-morbidity, multiple healthcare providers and cognitive impairment. This is a common combination that can often lead to excessive anticholinergic prescribing and patient harm. It also provides a timely reminder that many anticholinergic medications are available as over-the-counter medications without prescription, and these need to be considered when measuring anticholinergic burden.
